# The need to incorporate the impact of population ageing into the post-COVID-19 policy and planning reset in Low and Middle Income Countries

**DOI:** 10.1080/16549716.2021.1921351

**Published:** 2021-05-20

**Authors:** Geetesh Solanki, Gabrielle Kelly, Judith Cornell, Leon Geffen, Tanya Doherty

**Affiliations:** a Health Systems Research Unit, South African Medical Research Council; Health Economics Unit, University of Cape Town, Cape Town, South Africa; b Research Department, Samson Institute for Ageing Research, Cape Town, South Africa; c Institutional Development and Planning (Retired), Nelson Mandela School of Public Governance, University of Cape Town, Cape Town, South Africa; d Health Systems Research Unit, South African Medical Research Council, Cape Town, South Africa, School of Public Health, University of the Western Cape, Cape Town, South Africa

**Keywords:** Health policy, health systems, public health, ageing, COVID-19

## Abstract

The COVID-19 pandemic is likely to widen the health care demand-supply gap, especially in low- and middle-income countries (LMICs). The virus has had the greatest impact on older persons in terms of morbidity and mortality, and is occurring at a time of rapid population ageing, which is happening three times faster in LMICs than in high-income countries. Addressing the demand-supply gap in a post-COVID-19 era, in which resources are further constrained, will require a major ‘reset’ of the health system. In this article, we argue that the impact of ageing populations needs to be factored into the post-COVID-19 policy and planning reset including explicit, transparent prioritisation processes.

## Background

Regardless of the level of expenditure or methods of financing and service delivery adopted, no country can meet all the health care demands of its population. The gap between the demand for health care and the ability of health care systems to meet these demands in countries across the world, but particularly in Low- and Middle-Income Countries (LMICs), is well documented [1]. The COVID-19 pandemic is likely to widen this health care demand-supply gap [2]. On the supply side, the pandemic has had and will have a devastating long-term impact on the economic prospects of most countries [3]. On the demand side, COVID-19 has led to a huge increase in demand for resources, not only for health care, but also for other sectors such as social welfare and poverty relief programs. This widening of the supply–demand gap will require a ‘reset’ of existing policies and programs across countries globally, but particularly in LMICs such as South Africa.

This paper makes a case for factoring the impact of population ageing into this reset. Using South Africa as a case study, it examines the ageing trends in South Africa and their expected impact on health care demands, considers the progress made on policies, plans and programs to address the health and wellbeing of older persons, and sets out the challenges and actions required in the context of COVID-19.

## Ageing trends and impact on demand for health care

Increased longevity and decreasing fertility rates have resulted in ageing populations globally [4,5]. This change in population structure and the associated economic, social and health systems impacts are of growing concern to policymakers worldwide [6,7]. There is a strong link between a high prevalence of chronic disease, disability and ageing, and an increase in the care burden [8]. Poor health leads to lower quality of life and levels of well-being and higher levels of disability among older adults, especially among the poor [9]. Population ageing is happening three times faster in LMICs than in high-income countries and the relatively rapid pace of ageing in LMIC’s means that they will have a relatively shorter lead time in preparing for the demographic shift [8].

Research in South Africa forecast that between 2002 and 2022 the number and proportion of older adults in South Africa would increase from 3.3 million (7.2% of the population) to 5.7 million (9.1% of the population) [10]. The growth rate of the over-60 population is 2.7% per annum, nearly twice that of the population under 60 (1.4%). Between 2002 and 2022, while the overall population growth is expected to be 1.5% per annum, relative expected expenditure is expected to increase by 1.8% per annum. Ageing alone is therefore expected to increase relative expected expenditure by 0.3% per annum [10].

## Progress on policies and plans for older adults in South Africa

South Africa’s vision is to increase life expectancy from 61 to 70 years by 2030 [11]. South Africa adopted the Madrid International Plan of Action on Ageing in 2002 [12] and followed up by developing the South African Policy for Older Persons in 2005 [13], enacting the Older Persons Act and adopting the South African Plan of Action on Ageing in 2006 [14]. These are the only guiding policies and plans in place for addressing the issues related to older persons. Despite signing onto the WHO Global Strategy and Action Plan on Ageing and Health (2016–2020), and the WHO Decade of Health Ageing 2020–2030, no further related local plans or policies have to date been developed.

The South African Policy for Older Persons outlines a multi-sectoral response to the challenges of ageing and sets out four focus areas: (1) Social and economic development, including social protection; (2) Ensuring enabling and supportive living environments; (3) Protection from neglect and abuse; and (4) Advancing health and well-being into old age, which is the focus of this paper.

Progress on key action items for advancing health and well-being and ensuring that health services meet the specific needs of older persons as set out in the South African Plan on Ageing is summarised in Table 1. Progress on the key action items laid out in the plan was assessed based on a review of the health systems literature relevant to each item using relevant keywords on the MEDLINE, CINAHL and Scopus databases, as well as identifying policies that have been put in place by government that support the achievement of these goals. Where no appropriate evidence exists for an action item, this has been noted in Table 1.

**Table 1.** **Progress on action items from the South African plan of action on ageing** [14]

Action item in SA plan on ageing	Progress
Undertake research on older persons’ needs	Few studies published from 2006 to 2020: all part of broader international studies (e.g. WHO SAGE and INDEPTH HAALSI). None commissioned by the South African government.
Develop and review health budgets to ensure adequate funding is devoted to the provision of services (free access to older persons)	There is no evidence of this having taken place. A study in which key government and civil society stakeholders were interviewed found that mainstreaming of ageing issues has not taken place in part due to lack of appropriate budget allocations and resources within government departments [18].
Ensure in-service training for health professionals on health needs of older persons	Health professionals receive very little geriatric training and research has shown that health professionals lack the knowledge and skills to address older persons’ particular health needs [20].
Ensure appropriate and continuous training on ageing issues for community health workers	There is no evidence to establish whether this is occurring: unlikely given that no other initiatives have been implemented.
Ensure national coverage of comprehensive health services, including HIV and AIDS services for older adults	This objective is vague and difficult to measure. Primary health services (including care for NCDs and HIV and AIDS management) are widely available to the general population including older persons. However, specialised services are less available and accessible outside of urban areas.
Strengthen integrated geriatric services and training at all levels of the health care systems.	There is no evidence of efforts in this regard. In fact, existing research demonstrates high levels of dissatisfaction among older persons presenting at primary care facilities, low levels of quality of care, and a lack of trust in public healthcare professionals in both rural and urban settings [21,22].
Provide free health services to older persons, especially those with disability who are unable to meet the costs	This objective has been notionally achieved. PHC services, where available and accessible, are free to all older and elderly persons and secondary and tertiary healthcare services are free to all older people in receipt of a social grant (70% of persons over 60 receive social grants).
Develop and implement a strategy for the provision of safe traditional medicine	The Traditional Health Practitioners Act 22 of 2007 aims to regulate the provision of traditional health care to ensure safety and quality of services. A directorate of Traditional Medicine was also established within the NDoH and the Act establishes a Traditional Health Practitioners Council to manage the registration, training and conduct of traditional health practitioners and students. However, registration has been low due to poor knowledge and understanding, as well as suspicion by traditional health practitioners, and there is no code of conduct in place [23].
Provide comprehensive ophthalmic services for older persons	Ophthalmic services appear to be a low priority. They are poorly defined in health policy and there is no specific policy or financing model for eye health, resulting in poor service delivery and poor access to services, particularly in rural areas where there is inadequate equipment. There is a high prevalence of preventable age-related vision loss, pointing to inadequacy of eye-care services and eye health screening and promotion efforts [24–26]. The three leading causes of preventable blindness in older persons in South Africa (refractive error, cataracts and glaucoma) are addressed by national guidelines on refractive error screening for persons 60 years and older and on cataract surgery, as well as the National Guideline on the Prevention of Blindness in South Africa [27], but there is little information available on their implementation.
Provide comprehensive oral health services for older persons	Primary-level oral health services are free in terms of policy. Research has shown that there are significant barriers to use, including a lack of awareness of free services and accessibility challenges [28] and there are no programmes targeted specifically at older persons.
Subsidise the costs of assistive devices for older persons	Occupational therapists in health services can prescribe heavily subsidized basic assistive devices for older persons. However, knowledge of services, availability and waiting times act as constraints to access, and NGOs act to fill this gap.
Develop and implement a strategy for the management of chronic health conditions that are more prevalent in old age	The Draft NDoH Strategic Plan for the Prevention and Control of NCDs 2020–2025 [29] focuses on reducing and managing NCDs. The policy takes a life-course approach, but does not sufficiently focus on older persons’ needs
Implement national programmes on healthy lifestyles	Healthy lifestyle programmes targeting behavioural risk factors for NCDs have been developed as part of the National Strategic Plan for the Prevention and Control of NCDs. The *National Health Promotion Policy and Strategy 2015–19* [30] aims to improve longevity by promoting lifestyle change, creating supportive environments and developing personal skills for self-management of chronic conditions. This is achieved through community-based programmes and support groups run by health promoters. The Health for All health promotion tool has also been introduced in Primary Health Care facilities to promote primary and secondary health risk identification and mitigation. However, none of these programmes are targeted specifically at older persons and their age appropriateness is unclear.

NCD – non-communicable disease; NDoH – National Department of Health; NGO – Non-governmental organisation; WHO SAGE – World Health Organisation Study on Global AGEing and Adult Health; Health and Aging in Africa: A Longitudinal Study of an INDEPTH Community in South Africa.

As evident from Table 1, following on the adoption of the South African Plan of Action on Ageing in 2006 [14], there has been little in the way of research, policy development or action to implement the plan. While the government has developed strategic plans for the prevention and control of non-communicable diseases (NCD) in the general population [15], there is no focus on population ageing as a risk factor, or on the specific needs of older persons [15]. The latest draft NCD strategy for 2021 to 2026 promises to take the WHO’s life-course approach to prevention, management and control of NCDs, focusing on age-appropriate interventions [16]. However, at this stage the focus is strongly on prevention at an earlier age and discussion of the needs of older persons is limited to one line in the appendices: ‘older persons for example have special management needs related to their mobility that need to be addressed’ (page: 117).

The lack of progress in implementing the plans can arguably be attributed to a variety of factors including, amongst others, weak leadership and capacity [17], the co-ordination and accountability issues that arise as a result of the overlap in responsibilities across the two responsible departments in the South African context (Health and Social Development), and broader budgetary and fiscal constraints. More specifically, there appears to be a general lack of interest among policymakers in addressing ageing issues, which are de-prioritised in relation to what are seen as more pressing concerns (poverty, HIV/AIDS, youth dependency, etc.) [18]. Government structures and older persons’ organisations put in place to oversee the implementation of the Action Plan are weak and underfunded. To date, no dedicated resources or budgets appear to have been allocated to mainstreaming ageing issues across government departments or supporting the achievement of the goals outlined in the Older Persons Act or the Plan on Ageing. Implementation thus far has been left to local decentralised levels, with few incentives to increase efficiency or resource allocation for older adult health services [18].

## Impact of COVID-19 and action required in post-COVID-19 policy and planning reset

The COVID-19 pandemic will have a devastating impact on all spheres of life globally and has highlighted the extent to which the years of under-resourcing, compounded by mismanagement, corruption and incompetence have undermined the capacity of large portions of the public health sector in LMICs to deliver services to the populations that depend upon this sector [19].

The pandemic forced the South African Government to carry out a supplementary budget review in June 2020 and it is clear from the review that the pandemic will have a negative impact on the implementation of all health care policies, plans and programs [31]. The revised budget indicates that the pandemic has and will have a devastating impact on the economy and the capacity and/or willingness of government to increase the allocation of resources for health care, let alone retain current levels, will be limited [31]. In anticipation of the demands the pandemic will create for COVID-19 related health care and social support programs, the budget review entails a substantial re-allocation of budgeted expenditure from other programs (outside and within the health care sectors) towards COVID-19 related programs [31]. The re-allocation of resources from other programs towards COVID-19 efforts is likely to impact negatively on the other programs. However, it is unclear as to which programs will be worse affected and whether the impact on older adult programs will be more or less than the impact on other health programs.

On the other hand, the virus has already had the greatest impact on older persons in terms of morbidity and mortality, given their higher prevalence of NCDs and age-related loss of immunity [32]. It is likely to have an additional indirect impact on older persons’ health due to the disruption and redirection of resources within the health services to the management of COVID-19, lengthy social isolation and avoidance of health settings for fear of COVID infection [33]. In the longer term, whilst the elderly are to be prioritized for the receipt of COVID-19 vaccines [34] and the direct impact of the pandemic for the elderly may reduce, further constraints on resources as a result of the pandemic will exacerbate the impact of an ageing population on the demand for health care services.

Addressing the shortcoming of the current health care system as highlighted by the pandemic [35] in a post-COVID-19 era in which resources are going to be more constrained, will be ever more challenging and will require a major ‘reset’ of the health system. But this also presents an opportunity to ‘build back better’ so that the health care system addresses the health care needs of older adults more efficiently and appropriately and at the same time allows for more optimal use of scarce resources. The ‘reset’ should be used as an opportunity to ensure that going forward, epidemiological vulnerability is better managed by managing chronic disease more effectively, COVID-19 transmission vulnerability is reduced, health system vulnerability is limited, and the impact of control measures such as shielding and self-isolation, which may negatively impact on the health, mental health and wellbeing of older persons, is reduced.

The first WHO world report on ageing and health argues that there is an economic imperative for countries to adapt to shifts in the age structure in ways that minimize the expenditures associated with population ageing while maximizing the many contributions that older people make [36]. These societal contributions, especially informal caregiver time, are often not adequately considered in cost-effectiveness analyses of interventions focussed on older persons [37]. Country investments should consider a life-course perspective with the goal of ensuring a fair distribution of society’s resources. This does not require people in each age group to be treated exactly the same (given their different needs), but instead that they should be treated fairly throughout their life [38].

The elderly are rights-bearing citizens and important providers of care, especially child-care for low wage-earning working mothers. Investing resources to promote healthy ageing and enable people to preserve intrinsic capacity and functional ability for as long as possible improves quality of life, reduces dependency and related social and economic costs of ageing, as well as health systems costs as most interventions can take place at the primary care level, through delivery platforms such as community health workers where there are benefits for multiple age groups in one household.

More specifically, we propose the following ‘ageing’ related areas which should be addressed in the health care system planning reset.

We need to factor in the ageing of the population and the impact this will have on the demand for health care resources in all policy and planning initiatives.

We need to consider how early intervention in providing services to those in need of assistance with instrumental and basic activities of daily living, currently the domain of Social Development, can reduce the demand for secondary and tertiary services. This will require intersectoral collaboration between Health and Social Development with a clear strategy to ensure provision of effective long term care, as envisaged by the WHO policy framework for healthy ageing [36].

We need to recognise that some aspirational goals, e.g. provision of free and comprehensive health care services outside of primary care, are simply not going to be attainable in the short or medium term and the over-ambitious nature of these goals may have contributed to lack of progress in implementation of plans. While most countries operate explicit rationing for certain procedures, for example, organ transplant or kidney dialysis [39], LMICs rely largely on an implicit rationing model for health service delivery. This means that benefits are notionally open-ended, but health care services are implicitly rationed based on accessibility, availability of services, queues, waiting times and the discretion of health professionals. Waiting lists for specialist appointments and surgeries such as hip replacements can be so long that many patients will never receive these services [40]. This approach erodes the credibility of the health services, demoralises health workers who simply cannot deliver on what is promised with the resources provided and creates patient dissatisfaction, frustration and anger.

In dealing with this problem, we need to address the key question: ‘If all the health care services that are required/promised cannot be provided, what will be provided?’ Choices have to be made and priorities agreed upon as to what health services will be provided, for whom, and at what price and quality. To ensure that the choices we make are in the ‘best’ interests of society as a whole and in alignment with broader societal goals, we need to embark on a more explicit, transparent and public prioritisation process. Explicit priority-setting exercises are inherently complex and difficult to implement. However, the problem is not new and the experiences of countries that have grappled with these ethical, political and financial issues can be used as starting points and guides [41]. Countries can make use of the WHO ‘Best Buys’ – a set of population and individual level interventions identified by the WHO as affordable, feasible and cost-effective and which every country can implement to reduce the burden of NCDs [42].

In any prioritisation process, we need to recognise that we cannot afford to move towards more hospital-centred care. Rather, for older adults in LMIC contexts we should be looking at focusing on six key priority areas as summarised in Box 1. These priority areas are aligned with the World Health Organisation’s Action Plan for the Decade for Healthy Ageing 2020–2030 [43] and related WHO Guidelines on Integrated Care for Older People (ICOPE) [44], which put a strong emphasis on person-centered geriatric screening and assessment, care planning, health systems integration and community-level provision of care and support services to prevent, slow or reverse decline in the physical and mental capacities of older people. These recommendations are appropriate to both the context of COVID and the post-COVID era as the use of community health workers for outreach, screening and service delivery has benefits in terms of protecting vulnerable older persons during COVID and improving health care services for older persons more generally.

We would argue that the ‘reset’ required as a result of the pandemic will require policymakers to carry out major policy and program reviews and that this provides an ideal opportunity for reviewing the approach and the investment priorities related to older adult care and having that investment case evaluated. Equity and efficiency considerations would have to be major considerations in carrying out the reset and the priority areas that we have described offer the opportunity for efficiency gains which would be very attractive to policymakers. The timing for the consideration of these proposals is even more opportune within the South African context as the country is in the process of implementing major policy reform, in the form of national health insurance (NHI) towards the achievement of Universal Health Coverage (UHC) [45]. The NHI Bill entails the establishment of processes for Health Technology Assessment (HTA) and the necessary support systems and institutions to move towards a more explicit priority setting approach. Furthermore, the NHI will have a strong focus on strengthening primary level care including the establishment of a core package of services for different age groups [46].

This paper examines the need for the impact of ageing populations to be factored into the post-COVID-19 policy and planning reset, using South Africa as a case study. While specific circumstances may differ, we would argue that the challenges highlighted and the approach that we have advocated for South Africa would be relevant to many other LMICs.

**Box 1: Priority areas for improving the health of older persons in LMICs**

1. Placing greater emphasis on providing community care, primary care and palliative care for older adults. This may well require more restricted access to hospital care (and critical care in particular), with access limited to those cases where the prognosis is good.

2. Strengthening programs for prevention of disease and declines in capacity via:

- community prevention, health promotion and self-management campaigns that target older persons and consider their needs and interests.
- community and primary-level screening to facilitate early diagnosis and intervention.

3. Ensuring effective management of chronic conditions via:

- regular check-ups.
- necessary medication.
- a community-level focus incorporating home-based visits and support from appropriately trained community health workers in alignment with the WHO Global strategy and action plan on ageing and health and Action Plan for the Decade for Healthy Ageing [47].
- telemedicine programs [48].

4. Restructuring delivery of NCD and other services for older persons in the COVID context (given the need for older persons to be “shielded“) [49].
5. Mobilise and support civil society-based initiatives which empower people to take health into their own hands to mitigate the impact of the limited capacity of the public sector in many LMIC’s [50].

## Paper context

The COVID-19 pandemic is likely to widen the health care demand-supply gap. The virus has had the greatest impact on older persons and is occurring at a time of rapid population ageing. Addressing the demand-supply gap, in a resource-constrained post-COVID-19 era, requires a major ‘reset’ of health systems. The impact of ageing populations needs to be factored into the post-COVID-19 policy and planning reset including explicit, transparent prioritisation processes.
